# Supplementary material for: Failure to classically condition G. dorotocephala and S. mediterranea flatworms using historical and contemporary light-shock paradigms
Source: PLoS One. 2026 Sep 23;21(9):e0358756. doi: 10.1371/journal.pone.0358756 (PMC13600496; doi:10.1371/journal.pone.0358756)
Supplement: Supplementary file 1 [file pone.0358756.s006.docx]

PLOS One Manuscript ID: PONE-D-26-20336

Title: Failure to classically condition planarian flatworms

**Response to Reviewers**

We thank both reviewers for their careful reading of the manuscript and their constructive feedback. We have revised the manuscript accordingly and believe it is substantially improved by the reviewers’ comments. Below we address all comments (the reviewer comments are shown in italics, followed by our responses). Changes to the manuscript are indicated in bold in this letter and changes are tracked in the manuscript.

Response to Reviewer 1

We thank Reviewer 1 for their detailed and constructive feedback. Their comments identified several important issues with the organization, data presentation, and methodological transparency of the manuscript. We have made extensive revisions in response, which we believe have substantially improved the clarity and rigor of the paper. The major changes made in response to Reviewer 1 are:

- Reorganized the Results and Discussion into a single combined section to improve cohesion and reduce redundancy.
- Replaced protocol names (Jacobson, James) with plain-language labels ("historic" and "contemporary") used consistently throughout the manuscript.
- Added Table 2 directly comparing the historic and contemporary conditioning protocols.
- Restructured the old Figure 2 (now Figure 3) to compare conditioned response acquisition across species within a single protocol, and added a forest plot (New Figure 3C) to directly compare per-worm learning slopes against the historic reference rate.
- Moved the high/low responder figure to the supplement (Figure S2) and replaced the old Figure 1C with a cleaner group-average comparison.
- Added power analysis, Bayesian statistics (Bayes Factors, TOST), and Cohen's kappa to address concerns about sample size and interscorer agreement.
- Updated all figure captions to include species, sample sizes, and sufficient methodological detail for standalone interpretation.
- Standardized abbreviations (ITI, CS, UCS, CC, PC, UV) throughout the manuscript.
- Added references for planarian UV light research at Line 79.
- Updated error bars to 95% percentile bootstrap confidence intervals throughout, in place of SEM, to better reflect the skewed distribution of the data.

*The manuscript by Kelso et al presents attempts at replicating previous planarian conditioning experiments, ultimately concluding that none of the tested paradigms in either G. dorotocephala or S. mediterranea planarians induced robust conditioning. The experiments are well-designed and appear to be rigorously performed, though a more systematic approach would be helpful in some cases. I particularly appreciate the inclusion of computer vision to unbiasedly and quantitatively score conditioning responses. However, a lack of methodological details and confusing organization and data presentation make it difficult to determine if the data fully support the authors’ null conclusion.*

We thank the reviewer for this positive assessment of our replication attempt, experimental design, rigor, and computer vision pipeline. As described above, in line with the reviewer’s major comments about clarity, organization, and presentation, we have extensively revised the manuscript to make our findings clear.

*Introduction: Please be more specific about which planarian species have been used in the previous literature. Could differences in species explain any of the previous inconsistencies?*

We thank the reviewer for this comment and have amended the manuscript in two places to clarify the historically used species, and species used in our experiments. We have also amended the captions to include the species used in the respective figures.

1. Line 16-18: “These classical conditioning were typically conducted with *G. dorotocephala* flatworms”.

2. Lines 63-68: “We found no difference in conditioned response acquisition for any paradigm across two species: Girardia dorotocephala, which were historically used for psychological experiments, and S. mediterranea, which are commonly used in contemporary regenerative biology research. Finally, we found no difference in conditioning across three separate G. dorotocephala lineages from multiple locations across the United States (seeMethods for more details).”

The reviewer’s suggestion that species type could explain prior inconsistencies in the literature is interesting, but to the best of our knowledge this does not seem to be the critical factor in determining conditioning success. Perhaps the most notable failure to replicate came from using *Girardia dorotocephala* worms (Bennet and Calvin 1964), which were the same species that many groups found success in conditioning.

*There have been numerous examples demonstrating that different planarian species can show different types of behavioral reactions to the same stimuli (not just in conditioning experiments) and the authors state in the Results (line 109) that planarian species is important for the success of planarian learning. Thus, results across multiple species should not be lumped together without critical evaluation of the data first.*

We agree with the reviewer’s comment that species should not be lumped together, and have done the following to clarify that none of the subpopulations we attempted to condition showed a positive learning rate comparable to the historic rate:
 1. Restructured Figure 2A (the new Figure 3) to compare across species within a single experimental

protocol (similar to Figure S1).

2. Included statistics from Figure S1 reporting the learning rate for each subpopulation in the new

Figure 2B CC group; no subpopulation displayed learning comparable to the historic rate, and the

pooled CC group did not display learning comparable to the historic rate (lines 163-171)

The reviewer is correct in pointing out that the different species display different behavioral reactions to the same stimuli, and we want to reiterate that we confined the scope of our analysis to the two most relevant species, *Girardia dorotocephala* which was historically used for behavior, and *Schmidtea mediterranea,* which is used in contemporary regenerative biology (and many scientists we spoke to anecdotally have failed to condition).

*I find it very difficult to follow which methods are being used in various experiments. In the methods (and often in the SI figures), experimental paradigms are referred to by the original publication names (e.g. Jacobson et al, James et al). However, these distinctions are not clear in the Results text. It would make the paper much more accessible to either have plain-language methods names to refer to the different protocols or to make a schematic figure depicting what the different types of experiments refer to. Each experiment and figure should also explicitly describe which protocol is used. Addition of a figure or table comparing/contrasting the two main testing paradigms (Jacobson and James protocols) would be helpful.*

We agree with the reviewer that using the original paper names increases cognitive load, and have changed the manuscript to refer to each conditioning protocol in plain language as “historic” or “contemporary”. These names are established early in the manuscript and used consistently throughout to replace “Jacobson” and “James”.

Lines (47-50): “We refer to this protocol and our reproduction of it as the “historic” paradigm. We then tested a contemporary conditioning paradigm inspired by aversive conditioning in rodents (referred to as the “contemporary” paradigm), as well as a selection of ethologically-motivated alternative stimulus pairings (“variant” paradigms).”

We have also added a table that compares the historic and contemporary conditioning protocols (Table 2).

*The current organization of the Results is unclear and disjointed. In several instances it also seemed that the text descriptions did not match the referenced figures. Some concepts are only explained much later after they are first shown. For example, it seems that Results section 2.4 should come earlier with the description of Fig 1C as Fig 1C currently has no definition or low vs high responders, and that there is no separate color in Figure 1C referring to the combined data. Do the statistics in lines 69-74 match the combined data set or the separated data set shown in Fig 1C? It may be better to have Fig 1C show the original combined data and present the high vs low responder data in the Supplementary. Similarly, the caption for fig 3B does not seem to match the actual figure. The caption refers to multiple species but no separation is shown in the figure. There are also some aspects of data that appear in the figures but are not mentioned at all in the text (particularly in Fig 2A).*

We thank the reviewer for their critical reading of the text and have fixed the error in the caption of old figure 2 (now Figure 3) to reflect the contents of the figure. We agree that the presentation of our replication of the historic conditioning protocol (Fig 1C) was confusing, and have changed this figure to directly compare all worms for the CC versus PC protocols, moving the figure that separates low and high responders to supplemental Figure 2 (which reports the difference in baseline response to the CS). The statistics referring to Figure 1C now directly reflect the corresponding plot. We also agree that the section referring to high and low responders should come earlier and have reorganized the manuscript to reflect this (New Fig. 1 caption and lines 122-132) . To further clarify the organization we have moved Figure S3 to be part of Figure S2 (Figure S2C), which describes the differences in response to the CS across individuals.

*The authors perform experiments with two planarian species which I find to be very helpful and informative, but I find the presentation of these results to be unclear and misleading. In the Introduction, the authors should be more specific about which planarian species have been used in previous literature. Additionally, with the exception of Fig 2, which planarian species is used in the various experiments is not stated. Some of this data is provided in the methods (but not for all experiments) and should be more readily available in the results/figures.*

We thank the reviewer for their positive feedback on our use of two species, and have clarified which species are used in the introduction, and in the results:

Lines 16: “These classical conditioning procedures were typically conducted with *Girardia dorotocephala* flatworms and employed light and shock as the conditioned (CS) and unconditioned (UCS) stimuli, respectively.”

Lines 57-60: “In what follows, we describe our findings from attempting to classically condition \textit{G. dorotocephala} flatworms using (1) a historic protocol based on the work of Allan Jacobson , (2) a contemporary protocol based on the work of James et al. (2026), and (3) variant stimulus pairings informed by recent studies on planarian sensory capacities.

We have also updated the captions to indicate the species used (Fig. 1C, Fig. 2B-C, Fig. 3A-B), as well as additional methodological details and clarification (lines 48-52, 64, 71, 209). We have also updated the captions for 3 of the supplementary figures to accommodate the new panels, and further clarify the methodological details.

*In lines 115-116, the authors claim the different species and populations “showed indistinguishable levels of responding” referring to Fig 2A and Fig S1, but I find it hard to directly compare when it seems like not all the same conditions are tested in both species. I suggest separating out the data from multiple stimuli vs species differences. Fig S1 which directly compares the same conditions across the species and populations for the James et al paradigm is much more helpful than Fig 2A in this regard and should be moved to the main text. Fig 2A would be more convincing if averages and error bars were shown for the same stimulus (UV-shock) across the various populations. It is my assumption that the pale lines show example behaviors for a single worm and the dark lines are the averages, though this is not explained in the figure. But which species do the averages refer to?*

We thank the reviewer for the opportunity to clarify Figure 2A, and in line with their comments have split the first plot into two (New Figure 3A-B) to clearly show the conditioned response acquisition for each species for the same stimulus protocol. The pale lines in the new Figure 3A-B represent individual worm responses, and we have updated the figure caption to reflect this. In addition, in line with reviewer #2’s comment we have changed the error bars on the average of the plot to reflect a bootstrap of the 95% CI instead of the SEM. We have included an additional plot (Figure 3C) clarifying the comparison of individual slopes of conditioned response acquisition to the historic rate, which we believe helps communicate the main failure of our conditioning variants.

We respectfully believe that S1 should remain in the supplement. In line with the reviewer’s comment we have corrected the caption to refer correctly to New Figure 2B, and have included statistics of the individual worm slopes in comparison to the historic learning rate (New Figure S1)

*The various other testing stimuli seemed to only be tested in one species/population and this is not consistent, similarly making the data hard to interpret. I understand that testing all various combinations of stimuli/species is not possible but it would strengthen the paper to at least test every stimulus pair in* G. dorotocephala*, which appears to be the default species used for most experiments.*

We thank the reviewer for this constructive feedback and have clarified (through modification of New Figure 3) that each protocol was tested in *G. dorotocephala*. While we certainly agree it would be informative to thoroughly test conditioned response acquisition across every species and stimulus combination, we believe the pattern of results across the conditions we did test provides meaningful confidence that completing the full combinatorial space would be unlikely to change the overall conclusion. Across each combination of species (*G. dorotocephala* and *S. mediterranea*), stimulus pairing (UV-Shock, White-UV), and population (three independent *G. dorotocephala* lineages), we observed consistently negative or near-zero per-worm slopes, all of which fell significantly below the historic learning rate. The conditions we sampled were specifically chosen to vary the key dimensions of species, stimulus modality, and population origin, and the consistency of the pattern across all of these dimensions suggests that the untested combinations would be unlikely to yield a qualitatively different result. We acknowledge that this inference is not guaranteed, and we agree that a fully crossed design would be more definitive. However, given the resource constraints of exhaustive combinatorial testing and the consistency of the signal across the space we did sample, we believe the current dataset supports our conclusions while remaining appropriately cautious in their scope. Future work pursuing more ethologically relevant stimulus pairings would benefit from the fully crossed design the reviewer suggests.

*Many critical descriptions are missing from figure legends and captions throughout the manuscript, hindering interpretation of the data. The figures should be self-explanatory with the captions provided without requiring the reader to look for more information in the methods. For example, I find the legend in Fig 2A confusing as the source of the different G. dorotocephala populations is not included for all lines. There is also no distinction of low vs high UV stimulus which is referred to in the text. What is spring water referring to (only described in the methods and not in the actual results)? The figure legend is also small and hard to read.*

We thank the reviewer for critically reading the manuscript and have updated the captions to fully reflect the methods used in the main text (lines 163-171, Fig 2 caption). We agree with the reviewer that the format of the old Figure 2A is confusing, and have changed the data presentation (New Fig 3) for clarity (as stated above), corrected the captions to remove the erroneous labels, and increased the font size.

*Please clarify how you reconcile the claim in line 69 that there is 91.92% interscorer agreement with the data presented in section 2.5 that specific behavioral scores are not consistent across scorers? The 91.92% claim needs more context to understand what this is referring to. I think this may be due to the different experimental paradigms used, but this is not clearly presented.*

The reviewer’s intuition that different experimental paradigms mask the true interscorer agreement is correct, and we agree that this metric is provided without sufficient context. We claim in the manuscript that low interscorer agreement is a problem for quantifying the acquisition of the conditioned response, and maintain this is the case due to the biased distribution of conditioned response outcomes. In our data, zero (no response) is very common, so percent agreement is high even when raters don’t agree well on the uncommon but important outcome (here, 1). In the historic literature, percent agreement was not such a flawed metric because the distribution of 0’s and 1’s was more even. In our case, the high incidence of 0’s dominates the overall agreement rate and makes it look very high (the overall percent agreement was 91.9% (2945/3204), but on trials where at least one scorer marked a change (331/3204 trials), agreement was only 21.8% (72/331)). To be consistent with recent attempts at planarian conditioning (e.g., Prados et al. (2013)), we now include a Cohen’s kappa statistic in the main text (lines 231), reporting that our interscorer agreement was slightly above chance (Cohen’s kappa was 0.31 (P₀ = 0.92, Pₑ = 0.88)). These values and arguments are now given in lines 226-232.

*How was the sample size determined? Was any power analysis done? Sample sizes vary across experiments and some experiments are as low as n=5, which is very small. It is critical to show that these experiments were sufficiently powered to convincingly demonstrate that no conditioning occurred and null results are not just due to lack of power. Planarian behavioral data can be very noisy and it could be the small numbers used here could separate out signal to noise.*

We thank the reviewer for these comments on sample size and would like to clarify our experimental approach and conclusions. We have adjusted the wording in the manuscript to reflect these changes. Our primary goal was to test whether worms in our experimental setup would display learning rates on par with that demonstrated in the historic literature. Although the per-condition sample sizes (n=6-18 worms) are modest, the design was adequately powered to detect the conditioning effect reported by Jacobson et al. (1966). Historically the learning rate was reported as approximately +1.40 responses/block. Using the SD of per-worm slopes observed in our most variable condition (SM White-UV, SD = 1.21; Cohen's d = 1.16) and a one-sample t-test against this reference at alpha = 0.05, we estimate that n = 6 worms achieves 60% power and n = 18 achieves >99% power to detect an effect of this magnitude. Although 60% power is below the conventional 80% threshold, the mean slopes in these conditions were negative rather than merely small positive, making insufficient power an unlikely explanation for the null result.

Each population mean slope was significantly lower than Jacobson's +1.40/block reference both when the reference was treated as fixed (one-sample t-tests: p ≤ 0.009 in every condition; Cohen's d = −1.71 to −4.91) and when the uncertainty in Jacobson's own slope estimate was propagated using Welch's t-tests (p = 0.034, 0.009, and 0.012 for GD UV-Shock, GD White-UV, and SM White-UV, respectively; all 95% CIs on the slope difference excluded zero). We interpret these results as consistent with the conclusion that, under the protocols tested here, planaria did not acquire the conditioned response at a rate comparable to that originally reported by Jacobson et al.

We found our mean per-worm slopes were small and slightly negative in every condition: (New Fig. 3A-B) GD UV-Shock, -0.07 ± 0.07 (SEM) contractions/block (95% CI [-0.22, +0.08], n = 18); GD White-UV, -0.67 ± 0.44 (95% CI [-1.80, +0.47], n = 6); SM White-UV, -0.67 ± 0.49 (95% CI [-1.94, +0.60], n = 6). None reached significance for an upward trend (one-sided p ≥ 0.82 in each), and Bayesian one-sample t-tests using a JZS prior (r = √2/2) produced Bayes Factors that favored the no-trend hypothesis in all three conditions (BF₀₁ = 4.5, 3.1, and 3.5, respectively. This is moderate evidence for H₀ by Jeffreys' scale. For the best-powered condition (GD UV-Shock, n = 18), a two one-sided test (TOST) against a smallest effect size of interest (SESOI) of ±1.0 contraction/block was also significant (max p < 0.001), allowing us to positively conclude equivalence to no trend rather than simply failing to reject it. We set the SESOI at ±1.0 contraction/block, representing approximately 70% of Jacobson's reported effect, on the grounds that any conditioning effect smaller than this would not constitute meaningful replication of the historic result.

To be abundantly clear, we do not interpret these results as evidence that planaria are categorically incapable of being classically conditioned. Our claim is that with statistical power explicitly calibrated to the published Jacobson effect, we found no evidence of the historically reported learning rate.

The cohort of pulse-UV worms (n=5) acquired the response at a mean per-worm slope of +0.46 ± 0.20 SEM (n = 5, 95% CI [-0.10, +1.02]). Although the mean slope is positive and the confidence interval excludes Jacobson's published rate of +1.40 responses/block, the confidence interval also includes zero, which means we cannot draw a positive conclusion that any learning occurred. This ambiguity is compounded by two additional concerns. First, with n=5 the condition is substantially underpowered as the reviewer notes: using the observed SD of per-worm slopes from this condition (SD = 0.45), a one-sample t-test against zero at α = 0.05 achieves only approximately 50% power, meaning a real but modest learning effect could plausibly go undetected at this sample size. Second, and more importantly, the water pulse used as the conditioned stimulus in this paradigm introduces a physical perturbation that could itself elicit a reflexive response independent of conditioning, making interpretation of any positive trend difficult without additional controls. For these reasons (insufficient sample size and an unresolved physical confound) we have excluded this paradigm from the manuscript and plan to investigate this ethologically motivated stimulus pairing more thoroughly in future work with an appropriate sample size and suitable controls.

*I think the inclusion of shape analysis to better provide automated and quantifiable descriptions of behavior greatly improves the paper. But this could have been further explored as it was only used to analyze one experimental paradigm. If the authors have the video recordings for the other tested conditions, why not analyze those as well to see how the data compares with manual scoring? This would have been very interesting to see with the water pulse-UV tests that showed the closest to a real response (even with n only of 6).*

We thank the reviewer for their positive assessment of the computer vision pipeline and agree that this unbiased approach improves confidence in our negative results. We agree that this would make an excellent tool for examining changes in behavior due to repeated stimulus exposure (or conditioning) in future work. Because of the small sample sizes (as the reviewer mentioned) in the pulse-uv conditioning variant we have refrained from using our pipeline to attempt to draw conclusions about behavior during conditioning. The next steps for investigating whether conditioning variants produce any learning would be to repeat these experiments with an appropriate sample size to draw positive conclusions (specified above), and use our pipeline to extract and compare feature distributions. We believe this effort should be undertaken systematically and with odors or other chemogenic stimuli, as are typically used for conditioning in drosophila and *c. elegans*.

*Several of the abbreviations are not clearly defined and are not used in a standardized manner throughout the manuscript. Please be consistent in the terminology across different experimental paradigms.*

We thank the reviewer for attention to clarity and have standardized and defined abbreviations across the manuscript (intertrial interval = ITI, conditioned stimulus = CS, unconditioned stimulus = UCS, classical conditioning = CC, pseudoconditioning = PC, ultraviolet = UV).

*The initial paragraph at the beginning of the Results contains a lot of Conclusions/Discussion. Please revise this paragraph to provide the context for the work that will follow but move the discussion of the overall findings to Discussion. Alternatively, the results could become a combined Results and Discussion section.*

We thank the reviewer for their suggestion to combine the Results and Discussion. Upon rereading we agree this would greatly improve the manuscript’s cohesion, and we have restructured the main text results and discussion section accordingly.

*Line 79: add references to the planarian research on UV light that you are referring to.*

The following references on the planarian research on UV have been added (line 60):

Bray SR, Wyss LS, Chai C, Lozada ME, Wang B. Adaptive robustness through incoherent

signaling mechanisms in a regenerative brain. Cell Reports. 2024 Aug;43(8):114580.

Shettigar N, Chakravarthy A, Umashankar S, Lakshmanan V, Palakodeti D, Gulyani A.

Discovery of a body-wide photosensory array that matures in an adult-like animal and

mediates eye–brain-independent movement and arousal. Proceedings of the National

Academy of Sciences. 2021;118(20):e2021426118.

Shettigar N, Joshi A, Dalmeida R, Gopalkrishna R, Chakravarthy A, Patnaik S, et al.

Hierarchies in light sensing and dynamic interactions between ocular and extraocular sensory

networks in a flatworm. Science advances. 2017;3(7):e1603025.

Wyss LS, Bray SR, Wang B. Neuropeptide-mediated temporal sensory filtering in a

primordial nervous system. bioRxiv. 2024.

*What is the rationale behind classifying behaviors into turns and contractions?*

We manually classified conditioned response behavior into turns and contractions to remain consistent with the scoring method from the historic literature (see Corning and Riccio (1970), Jacobson (1967), and Corning and Kelly (1973)). More recent studies (as cited) from 2013 and 2026 also use these measures. Historically, turns and contractions were assessed both separately and pooled. We have included an additional citation to make this clear in the main text (lines 18-21) and methods (lines 432-435).

*Could a turn and a contraction both be evidence of a response and thus grouping them together could give a more reliable measure?*

The reviewer brings up an insightful point, suggesting that grouping both turns and contractions may provide a more reliable measure of conditioned response acquisition. While it is possible that both turns and contractions provide equal evidence of conditioned response acquisition, we believe there is rich behavioral nuance in planarian behavior during conditioning. This intuition from observing behavior from many worms in different contexts motivated us to develop the computer vision pipeline to decrease bias in our conditioned response scoring. In the Jacobson studies cited in the paper, it was determined that contractions, but not turns, are the reliable conditioned response. That trend is also seen in the recent James et al. paper. Furthermore, at least for our contemporary replication, the number of turns vastly outnumbers the number of contractions, so pooling them makes little difference and does not change the results (i.e., stacking two flat lines does not produce a sloped line).

*What is the purpose of pre-coating the troughs with mucus from non-experimental planarians?*

The purpose of pre-coating the troughs with mucus from non-experimental planarians was to follow historical procedures as faithfully as possible. While the specific Jacobson et al. (1966) paper did not explicitly report a pre-training slime period, multiple reports suggest that slime from non-experimental animals is critical for acclimatization of experimental worms to the assay trough, and its absence affect behavior. We thank the reviewer for bringing up this methodological detail, and upon rereading we see that it was not properly historically justified. We have updated the manuscript to reflect this change (lines 454-459).

Response to Reviewer 2

We thank Reviewer 2 for their careful reading of the manuscript and their constructive suggestions regarding the clarity of our methodology, the scope of our conclusions, and the presentation of our figures. We have revised the manuscript substantially in response to their comments. The major changes made in response to Reviewer 2 are:

- Narrowed the scope of claims in the abstract, results, and discussion to reflect only the two species tested (*G. dorotocephala* and *S. mediterranea*) and the specific protocols used, removing broad generalizations not supported by the data.
- Added Table 2 to the main text directly comparing the historic (Jacobson) and contemporary (James) conditioning protocols, addressing the lack of a clear methodological comparison.
- Clarified the rationale for selecting each conditioning variant paradigm, grounding stimulus choices in recent literature on planarian sensory capacity.
- Expanded the discussion (now a combined results and discussion section) to address the distinction between failure of specific protocols and the broader question of planarian learning capacity.
- Updated figure colors to remove red/green combinations and standardized color coding across all main figures.
- Added a time axis to Figure 1B and sample sizes to Figure 1C and 1D.
- Reprinted the original Jacobson figure in the supplement (Figure S3) for direct comparison.
- Replaced frame numbers with seconds on x-axes in the old Figure 3 (now Figure 2) for interpretability.
- Added interpretive caption text for the z-score plots in Figure 2A (formerly Figure 3).
- Removed the pulse-UV condition from the manuscript, noting that this paradigm warrants further investigation at a larger sample size in future work.
- Updated error bars to 95% percentile bootstrap confidence intervals throughout to reflect the non-normal distribution of the data.

*According to the authors, this work aimed to revisit the question of whether freshwater flatworms (planarians) can learn via classical conditioning, which was the basis of controversial historical studies wherein memory of such learned tasks was transferred via cannibalism to naïve planarians. Most of the authors’ experiments use the species* Girardia dorotocephala*, which was the species used in the classical experiments; the authors also do very limited tests using the species* Schmidtea mediterranea*, which is the most popular species for regeneration studies in the field today.* *While revisiting and trying to reproduce historical studies is interesting and valuable, the manuscript in its current form fails to clearly present the historical methodology and results as well as the authors’ own methodology and results.*

We thank the reviewer for their positive assessment of the value of our study, and their interest in our findings. Upon rereading the manuscript we agree that the organization is unclear, and in line with the reviewer’s comments have reordered the flow of the paper to better communicate the historical methodology, our contemporary methodological details, and our null results.

*The way the manuscript is written, it appears that the authors tried to closely reproduce the methodology of Jacobson et al., (1967). It is a bit unclear why they chose that specific protocol nor is a clear comparison provided that would allow a reader to assess the similarity of approaches; a detailed comparative table in the main text would be helpful. It would also be helpful here to bring in the other conditioning paradigm that they copied later in the study, from James et al, 2025, for comparison. The overall lack of clarity and precision in the writing and missing details in the methodology make the assessment of the presented findings challenging.*

We thank the reviewer for the opportunity to clarify why we selected this protocol to replicate, and have changed the main text to reflect this (lines 45-47 and the new Table 2). Per the suggestion of the reviewer, we have included a table in the main text that directly compares the historical and contemporary methodologies.

We have amended the organization of the paper to better communicate our findings. The main changes we made were:

(1) Defined the protocols we used in plain language at the beginning of the manuscript (“contemporary” versus “historical”) and clearly state when each protocol was used in the results and figure captions.
(2) Reorganized the figures to better compare our results from the historic and contemporary protocols (swapped figures 2 and 3).
(3) Moved the section reporting the effects of individual differences on conditioning up to Figure 1 so that we discuss one protocol at a time.
(4) Changed Figure 1 to show the group averages rather than individual differences.
(5) Reorganized the supplementary figures to group all reporting of initial responses to the conditioned stimulus in a single figure (S2)
(6) Reprinted the original Jacobson figure in the supplement.

*The number of replicates in several experiments is too small to draw conclusions.*

We thank the reviewer for the opportunity to clarify the claims made with our data from the particularly small sample size populations. The sample sizes (even the modest ones of n = 6) were sufficient to conclude that the learning rate of our worms was significantly lower than that of the historic paradigm. Using the SD of per-worm slopes observed in our most variable condition (SM White-UV, SD = 1.21; Cohen's d = 1.16) and a one-sample t-test against this reference at alpha = 0.05, we estimate that n = 6 worms achieves 60% power and n = 18 achieves >99% power to detect an effect of this magnitude. While 60% power is below the conventional 80% threshold, the mean slopes in these conditions were negative rather than merely small positive, making insufficient power an unlikely explanation for the null result (lines 83-93, 163-171, 216-222). We agree that this conclusion was not made clear in the original version of the paper and have amended the language in the results to reflect the primary goal of our manuscript, which was to test whether we could replicate anything close to this learning rate (+1.4 responses/training block). We have included an additional figure (new Figure 3C) to clarify this comparison of learning rates, and show that our data indicate that no population met this learning rate criterion. Upon rereading the manuscript we agree that the pulse-uv conditioning paradigm should be further investigated in a separate study, as the confounds of the physical water pulse did not allow us to claim that the learning rate was significantly different than the historic rate.

*Besides trying to use the original paradigm, the authors also tried different cue combinations and training paradigms; it is unclear how they chose the training paradigms. How do we know whether learning presumably failed due to the ‘wrong training paradigm’ or because the organism is unsuitable for conditioning? – It seems like this question would warrant a more extensive discussion.*

We thank the reviewer for the opportunity to elaborate on how we chose the additional training paradigms, and have changed the manuscript to more clearly communicate this (lines 145-152). Recent literature on planarian sensory capacity reveals that UV light is a highly salient stimulus that produces different behavioral responses at high and low intensities (Shettigar et al. 2017, Shettigar et al. 2021, Bray et al. 2024, Wyss et al. 2024). Planarians have UV-sensitive extraocular opsins distributed across their bodies that mediate a rapid contraction in response to high-amplitude UV light exposure. Low-amplitude and slowly modulating UV light evokes a more subtle response, and is suitable as conditioned stimuli.We selected two new stimulus pairings (CS and US): (1) white light and high-amplitude ultraviolet (UV) light, and (2) low-amplitude UV light and shock. We suspected that planarians may display implicit biases for detecting and associating these sets of stimuli, making it easier to elicit a robust conditioned response.

The reviewer’s question also raises an excellent point, which we further clarify in the manuscript– we do not believe that planaria are unsuitable for conditioning, but rather that the protocols that historically elicited robust conditioning do not appear to do so in our hands. We have added to the discussion to make this point abundantly clear (lines 179-183, 484-498), and thank the reviewer for this comment to better scope the claims of the manuscript.

*It is recommended to adapt the writing to describe in detail the actual experiments conducted and their results and eliminate broad inferences and claims of generality of these findings that are not supported by the data. Moreover, having tested only two species with a limited number of cues, the authors should refrain from making broad statements and conclusions about the generality of their findings applying to all planarian species and implications regarding memory transfer which was not part of this work. Abstract and discussion need to be edited accordingly. In this regard, both title and abstract should clearly list the species names and the framework of conditioning should be detailed in the abstract.*

We thank the reviewer for their comments on the scope of claims, and have removed statements making claims beyond what our data reflect:

Removed: “If all planarian experiments obtain conditioned responses as unreliable as ours, then planarians seem a poor organism with which to investigate these questions.”

Inserted (Lines 305-308): “ We believe our efforts do not reflect on the widespread ability of planarians to learn in any situation, but conclude that we were unable to specifically elicit classical conditioning in the historically championed species *G. dorotocephala* or commonly used species *S. mediterranea* using historic and contemporary conditioning paradigms”

Inserted (Lines 50-52): “In all cases, we were unable to find evidence for classical conditioning in *Girardia dorotocephala* or *Schmidtea mediterranea* planarians”

Modified the abstract to specify the species: “Our results cast doubt on the suitability of historic conditioning protocols for *Girardia dorotocephala* and *Schmidtea mediterranea* planarians, and we encourage future research to pursue more ethologically relevant stimuli in pursuit of conditioning robust enough to investigate the molecular basis of learning.”

*Fig1: It would be helpful to provide a time axis for panel B explaining the training paradigm, list number of worms tested in C and D, explain how historical data was adapted (why was it not reprinted?)*

Per the reviewer’s comments we have added a time axis to panel B to show the training paradigm, which is further explained in the supplementary section on the historic conditioning protocol. We added the number of worms tested to the figure in panels C and D. The historical data was simply reprinted for visual clarity of comparison with our data, and it is now reprinted in its original form in the supplement (New Figure S3).

*How does your apparatus compare to the original one?*

Our apparatus is as close replication of the McConnell’s original devices as we could create with modern materials (McConnell, Cornwell, and Clay (1960) “An Apparatus for Conditioning Planaria”). McConnell’s device was a single thin plastic lane with terminal electrodes; our trough is made of similar thin plastic lanes, except that we simply have more of them in parallel so that we could train multiple (but still isolated) worms at once, recorded with an overhead camera. We do not replicate the exact proportions of his device because those details are not provided. We thank the reviewer for noting this and we have updated the Methods section (lines 443-449).

*Explain the definition of low and high responders in the caption.*

Per the reviewer’s comment we define low and high responders in the caption. In addition, in line with Reviewer #1’s comment, we have moved the original Figure 1C plot of the high and low responders to the supplement (Figure S2) and replaced this panel with the average of all responders for CC and PC. We discuss high and low responders in the results section (section 2.2 generally, lines 100-110 specifically). To address the concerns here, we define low and high responders in the caption in the supplemental figure.

*Please avoid using red and green colors together in plots. The color coding also seems to vary randomly across the main figures.*

We have changed the color scheme of the plots that contained red and green to align with the reviewer’s comment, and have modified the global color scheme (particularly panel B and C in New Figure 2) to be consistent between Figures 1, 2, and S2 (the colors for CC and PC conditions.)

*Fig.2: Labels are too small. Panel A – What is shown? Individual worm responses are thin lines and broad lines with error bars are means with SEM? Is the data normally distributed?*

We thank the reviewer for their questions on Figure 2, and have modified it to improve clarity. In the original figure, panel A showed the conditioned response acquisition for three species x protocol combinations. The light traces were individual worms and the dark line was the average with SEM error bars. As many responses were zeros, the data are not normally distributed, which is why we included the light traces to show the individual conditioned responses across training. We thank the reviewer for noting this and we have updated the error bars in New Figure 3A-B to 95% percentile bootstrap CIs on the daily mean (10,000 resamples), because the per-trial, per-worm score distributions are heavily right-skewed (mostly zero with a long upper tail). We have also updated the error bars to 95% percentile bootstrap CIs in Figure 1C, Figure S1, New Figure 2B, and Figure S4, as the data here are also not normally distributed.

*What is being summarized ? The response to the same stimulus, across species? If so, why aggregate across species?*

This figure summarizes the rate of the conditioned response acquisition across two new conditioning protocols that use UV light as the conditioned and unconditioned stimulus, respectively. We thank the reviewer for this question and agree that this visualization is unclear, and have therefore split the first panel into two so that each plot shows the conditioned response acquisition for a single conditioning protocol, rather than showing all protocols x species on one plot (New Figure 3A,B). In addition, (in line with reviewer #1’s comments) we have removed the pulse-uv condition (prior panel B) because we believe this protocol should be investigated in greater depth in subsequent work, and does not contribute to the manuscript meaningfully at this sample size.

*Fig.3: It would be helpful to give each figure element a panel letter for clarity. For the traces, I suggest using time instead of frame number for the x-axis as frame is arbitrary.*

Per the reviewer’s suggestion we have replaced frames with seconds for better interpretability, and added an interpretation of the traces to the caption.

*Briefly explain in the caption what the interpretation of the z-score plots is for the data shown.*

We have added a brief interpretation of the z-score plots shown in Panel A of this figure (New Figure 2).

“Traces show z-scored feature values for an example worm during CS-on (red) and ITI (blue) periods; heatmaps show corresponding feature distributions. Increasing area with stable perimeter indicates lateral body spread; spikes in circularity with decreasing perimeter indicate contraction.”

*It would be helpful to have a schematic showing the James et al. protocol for context for B and C. Again, it seems like the data here was aggregated across species. Why?*

We thank the reviewer for the opportunity to clarify that panel B shows the average for the contemporary conditioning protocol, aggregated across species that individually do not show learning rates comparable to the historic learning rate in Jacobson et al. We have included these statistics in the caption of Figure S1. This figure aggregates across species because we wanted to provide a visual comparison with our KL divergence metric (panel C), and all populations showed a similar trend in failing to acquire the conditioned response.
