# Supplementary material for: Failure to classically condition G. dorotocephala and S. mediterranea flatworms using historical and contemporary light-shock paradigms
Source: PLoS One. 2026 Sep 23;21(9):e0358756. doi: 10.1371/journal.pone.0358756 (PMC13600496; doi:10.1371/journal.pone.0358756)
Supplement: Supplementary file 2 [file pone.0358756.s007.docx]

PLOS One Manuscript ID: PONE-D-26-20336

Title: Failure to classically condition planarian flatworms (now updated to “Failure to classically condition *G. dorotocephala* and *S. mediterranea* flatworms using historical and contemporary light-shock paradigms”)

**Response to Reviewers**

We thank both reviewers for their continued feedback and have revised the manuscript in line with the remaining comments. Below we address all comments (the reviewer comments are shown in italics, followed by our responses). A PDF of the manuscript with tracked changes shown is attached.

Response to Reviewer 1

*“I appreciate the authors' efforts to incorporate the reviewers' feedback and think the paper is much improved. The authors have appropriately narrowed the scope of their conclusions to match the data presented; however, I believe it is still necessary to change the title accordingly, as previously mentioned by Reviewer 2, to make it clear which species were tested and that this failure is specific to specific testing protocols. Otherwise, I believe the manuscript is suitable for publication.”*

We thank the reviewer for this comment to further clarify the scope of the paper, and have updated the title to include the planarian species and conditioning paradigms used.

Response to Reviewer 2

*“As mentioned previously, the title should explicitly name the planarian species that were tested.”*

We thank the reviewer for their agreement to change the title, and have done as such (see above). We agree that this further clarifies the scope of the manuscript and claims.

*“Please remove bioRxiv articles from the citations that have not yet undergone peer-review.”*

We thank the reviewer for this comment and recognize the importance of restricting citations to published, peer-reviewed research. We have removed the BioRxiv citations of the Wyss et al. paper and have replaced the Samuel et al. citation with the peer-reviewed version published in 2026.

*“Remove lines 291-292 from the Discussion "Because of our failure to demonstrate conditioned behavior, we were not able to address questions of molecular transfer of memory between planarians in any way. " - a study of memory transfer was not within the scope of this work.”*

We understand the reviewer’s concern that this sentence distracts from the experiments presented in the paper, and upon rereading agree it isn’t suitable in the manuscript. The sentence has been removed.
