# Supplementary material for: Failure to classically condition G. dorotocephala and S. mediterranea flatworms using historical and contemporary light-shock paradigms
Source: PLoS One. 2026 Sep 23;21(9):e0358756. doi: 10.1371/journal.pone.0358756 (PMC13600496; doi:10.1371/journal.pone.0358756)
Supplement: Peer review history

# Author comment: Author response to Decision Letter 0

**DOI:** 10.1371/journal.pone.0358756.r001

**Transfer Alert**

This paper was transferred from another journal. As a result, its full editorial history (including decision letters, peer reviews and author responses) may not be present.

27 Apr 2026

# Peer review: Decision Letter 0

**DOI:** 10.1371/journal.pone.0358756.r002

**Authors:** Maria Y Pakharukova

2 Jun 2026

Dear Dr. Kelso,

Thank you for submitting your manuscript to PLOS ONE. After careful consideration, we feel that it has merit but does not fully meet PLOS ONE’s publication criteria as it currently stands. Therefore, we invite you to submit a revised version of the manuscript that addresses the points raised during the review process.

Please submit your revised manuscript by Jul 17 2026 11:59PM. If you will need more time than this to complete your revisions, please reply to this message or contact the journal office at plosone@plos.org. When you're ready to submit your revision, log on to https://www.editorialmanager.com/pone/ and select the 'Submissions Needing Revision' folder to locate your manuscript file.

- A letter that responds to each point raised by the academic editor and reviewer(s). You should upload this letter as a separate file labeled 'Response to Reviewers'.
- A marked-up copy of your manuscript that highlights changes made to the original version. You should upload this as a separate file labeled 'Revised Manuscript with Track Changes'.
- An unmarked version of your revised paper without tracked changes. You should upload this as a separate file labeled 'Manuscript'.

If you would like to make changes to your financial disclosure, please include your updated statement in your cover letter. Guidelines for resubmitting your figure files are available below the reviewer comments at the end of this letter.

If applicable, we recommend that you deposit your laboratory protocols in protocols.io to enhance the reproducibility of your results. Protocols.io assigns your protocol its own identifier (DOI) so that it can be cited independently in the future. For instructions see: https://journals.plos.org/plosone/s/submission-guidelines#loc-laboratory-protocols. Additionally, PLOS ONE offers an option for publishing peer-reviewed Lab Protocol articles, which describe protocols hosted on protocols.io. Read more information on sharing protocols at https://plos.org/protocols?utm_medium=editorial-email&utm_source=authorletters&utm_campaign=protocols.

As the corresponding author, your ORCID iD is verified in the submission system and will appear in the published article. PLOS supports the use of ORCID, and we encourage all coauthors to register for an ORCID iD and use it as well. Please encourage your coauthors to verify their ORCID iD within the submission system before final acceptance, as unverified ORCID iDs will not appear in the published article. *Only*  the individual author can complete the verification step; PLOS staff *cannot*  verify ORCID iDs on behalf of authors.

We look forward to receiving your revised manuscript.

Kind regards,

Maria Y Pakharukova, Ph.D., D.Sc.

Academic Editor

PLOS One

Journal requirements:

When submitting your revision, we need you to address these additional requirements.

1. Please ensure that your manuscript meets PLOS ONE's style requirements, including those for file naming. The PLOS ONE style templates can be found at

https://journals.plos.org/plosone/s/file?id=wjVg/PLOSOne_formatting_sample_main_body.pdf and

https://journals.plos.org/plosone/s/file?id=ba62/PLOSOne_formatting_sample_title_authors_affiliations.pdf

2. Thank you for stating the following financial disclosure:

“This research was funded by a Polymath Award from Schmidt Sciences.

https://www.schmidtsciences.org/grantee/sam-gershman/”

Please state what role the funders took in the study.  If the funders had no role, please state: "The funders had no role in study design, data collection and analysis, decision to publish, or preparation of the manuscript."

If this statement is not correct you must amend it as needed.

Please include this amended Role of Funder statement in your cover letter; we will change the online submission form on your behalf.

3. In the online submission form, you indicated that [Insert text from online submission form here].

All PLOS journals now require all data underlying the findings described in their manuscript to be freely available to other researchers, either 1. In a public repository, 2. Within the manuscript itself, or 3. Uploaded as supplementary information.

This policy applies to all data except where public deposition would breach compliance with the protocol approved by your research ethics board. If your data cannot be made publicly available for ethical or legal reasons (e.g., public availability would compromise patient privacy), please explain your reasons on resubmission and your exemption request will be escalated for approval.

4. We notice that your supplementary figures are included in the manuscript file. Please remove them and upload them with the file type 'Supporting Information'. Please ensure that each Supporting Information file has a legend listed in the manuscript after the references list.

If the reviewer comments include a recommendation to cite specific previously published works, please review and evaluate these publications to determine whether they are relevant and should be cited. There is no requirement to cite these works unless the editor has indicated otherwise.

[Note: HTML markup is below. Please do not edit.]

Reviewers' comments:

Reviewer's Responses to Questions

**Comments to the Author**

1. Is the manuscript technically sound, and do the data support the conclusions?

Reviewer #1: Partly

Reviewer #2: No

**********

2. Has the statistical analysis been performed appropriately and rigorously?

Reviewer #1: Yes

Reviewer #2: No

**********

3. Have the authors made all data underlying the findings in their manuscript fully available?

The PLOS Data policy

Reviewer #1: Yes

Reviewer #2: Yes

**********

4. Is the manuscript presented in an intelligible fashion and written in standard English?

Reviewer #1: Yes

Reviewer #2: Yes

**********

Reviewer #1: The manuscript by Kelso et al presents attempts at replicating previous planarian conditioning experiments, ultimately concluding that none of the tested paradigms in either G. dorotocephala or S. mediterranea planarians induced robust conditioning. The experiments are well-designed and appear to be rigorously performed, though a more systematic approach would be helpful in some cases. I particularly appreciate the inclusion of computer vision to unbiasedly and quantitatively score conditioning responses. However, a lack of methodological details and confusing organization and data presentation make it difficult to determine if the data fully support the authors’ null conclusion.

Major comments:

Introduction: Please be more specific about which planarian species have been used in the previous literature. Could differences in species explain any of the previous inconsistencies? There have been numerous examples demonstrating that different planarian species can show different types of behavioral reactions to the same stimuli (not just in conditioning experiments) and the authors state in the Results (line 109) that planarian species is important for the success of planarian learning. Thus, results across multiple species should not be lumped together without critical evaluation of the data first.

I find it very difficult to follow which methods are being used in various experiments. In the methods (and often in the SI figures), experimental paradigms are referred to by the original publication names (e.g. Jacobson et al, James et al). However, these distinctions are not clear in the Results text. It would make the paper much more accessible to either have plain-language methods names to refer to the different protocols or to make a schematic figure depicting what the different types of experiments refer to. Each experiment and figure should also explicitly describe which protocol is used. Addition of a figure or table comparing/contrasting the two main testing paradigms (Jacobson and James protocols) would be helpful.

The current organization of the Results is unclear and disjointed. In several instances it also seemed that the text descriptions did not match the referenced figures. Some concepts are only explained much later after they are first shown. For example, it seems that Results section 2.4 should come earlier with the description of Fig 1C as Fig 1C currently has no definition or low vs high responders, and that there is no separate color in Figure 1C referring to the combined data. Do the statistics in lines 69-74 match the combined data set or the separated data set shown in Fig 1C? It may be better to have Fig 1C show the original combined data and present the high vs low responder data in the Supplementary. Similarly, the caption for fig 3B does not seem to match the actual figure. The caption refers to multiple species but no separation is shown in the figure. There are also some aspects of data that appear in the figures but are not mentioned at all in the text (particularly in Fig 2A).

The authors perform experiments with two planarian species which I find to be very helpful and informative, but I find the presentation of these results to be unclear and misleading. In the Introduction, the authors should be more specific about which planarian species have been used in previous literature. Additionally, with the exception of Fig 2, which planarian species is used in the various experiments is not stated. Some of this data is provided in the methods (but not for all experiments) and should be more readily available in the results/figures. In lines 115-116, the authors claim the different species and populations “showed indistinguishable levels of responding” referring to Fig 2A and Fig S1, but I find it hard to directly compare when it seems like not all the same conditions are tested in both species. I suggest separating out the data from multiple stimuli vs species differences. Fig S1 which directly compares the same conditions across the species and populations for the James et al paradigm is much more helpful than Fig 2A in this regard and should be moved to the main text. Fig 2A would be more convincing if averages and error bars were shown for the same stimulus (UV-shock) across the various populations. It is my assumption that the pale lines show example behaviors for a single worm and the dark lines are the averages, though this is not explained in the figure. But which species do the averages refer to?

The various other testing stimuli seemed to only be tested in one species/population and this is not consistent, similarly making the data hard to interpret. I understand that testing all various combinations of stimuli/species is not possible but it would strengthen the paper to at least test every stimulus pair in G. dorotocephala, which appears to be the default species used for most experiments.

Many critical descriptions are missing from figure legends and captions throughout the manuscript, hindering interpretation of the data. The figures should be self-explanatory with the captions provided without requiring the reader to look for more information in the methods. For example, I find the legend in Fig 2A confusing as the source of the different G. dorotocephala populations is not included for all lines. There is also no distinction of low vs high UV stimulus which is referred to in the text. What is spring water referring to (only described in the methods and not in the actual results)? The figure legend is also small and hard to read.

Please clarify how you reconcile the claim in line 69 that there is 91.92% interscorer agreement with the data presented in section 2.5 that specific behavioral scores are not consistent across scorers? The 91.92% claim needs more context to understand what this is referring to. I think this may be due to the different experimental paradigms used, but this is not clearly presented.

How was the sample size determined? Was any power analysis done? Sample sizes vary across experiments and some experiments are as low as n=5, which is very small. It is critical to show that these experiments were sufficiently powered to convincingly demonstrate that no conditioning occurred and null results are not just due to lack of power. Planarian behavioral data can be very noisy and it could be the small numbers used here could separate out signal to noise.

I think the inclusion of shape analysis to better provide automated and quantifiable descriptions of behavior greatly improves the paper. But this could have been further explored as it was only used to analyze one experimental paradigm. If the authors have the video recordings for the other tested conditions, why not analyze those as well to see how the data compares with manual scoring? This would have been very interesting to see with the water pulse-UV tests that showed the closest to a real response (even with n only of 6).

Several of the abbreviations are not clearly defined and are not used in a standardized manner throughout the manuscript. Please be consistent in the terminology across different experimental paradigms.

Minor comments:

The initial paragraph at the beginning of the Results contains a lot of Conclusions/Discussion. Please revise this paragraph to provide the context for the work that will follow but move the discussion of the overall findings to Discussion. Alternatively, the results could become a combined Results and Discussion section.

Line 79: add references to the planarian research on UV light that you are referring to.

What is the rationale behind classifying behaviors into turns and contractions? Could a turn and a contraction both be evidence of a response and thus grouping them together could give a more reliable measure?

What is the purpose of pre-coating the troughs with mucus from non-experimental planarians?

Reviewer #2: According to the authors, this work aimed to revisit the question of whether freshwater flatworms (planarians) can learn via classical conditioning, which was the basis of controversial historical studies wherein memory of such learned tasks was transferred via cannibalism to naïve planarians. Most of the authors’ experiments use the species Girardia dorotocephala, which was the species used in the classical experiments; the authors also do very limited tests using the species Schmidtea mediterranea, which is the most popular species for regeneration studies in the field today.

While revisiting and trying to reproduce historical studies is interesting and valuable, the manuscript in its current form fails to clearly present the historical methodology and results as well as the authors’ own methodology and results. The way the manuscript is written, it appears that the authors tried to closely reproduce the methodology of Jacobson et al., (1967). It is a bit unclear why they chose that specific protocol nor is a clear comparison provided that would allow a reader to assess the similarity of approaches; a detailed comparative table in the main text would be helpful. It would also be helpful here to bring in the other conditioning paradigm that they copied later in the study, from James et al, 2025, for comparison. The overall lack of clarity and precision in the writing and missing details in the methodology make the assessment of the presented findings challenging. The number of replicates in several experiments is too small to draw conclusions. Besides trying to use the original paradigm, the authors also tried different cue combinations and training paradigms; it is unclear how they chose the training paradigms. How do we know whether learning presumably failed due to the ‘wrong training paradigm’ or because the organism is unsuitable for conditioning? – It seems like this question would warrant a more extensive discussion.

It is recommended to adapt the writing to describe in detail the actual experiments conducted and their results and eliminate broad inferences and claims of generality of these findings that are not supported by the data. Moreover, having tested only two species with a limited number of cues, the authors should refrain from making broad statements and conclusions about the generality of their findings applying to all planarian species and implications regarding memory transfer which was not part of this work. Abstract and discussion need to be edited accordingly. In this regard, both title and abstract should clearly list the species names and the framework of conditioning should be detailed in the abstract.

Main figures:

Fig1: It would be helpful to provide a time axis for panel B explaining the training paradigm, list number of worms tested in C and D, explain how historical data was adapted (why was it not reprinted?) How does your apparatus compare to the original one? Explain the definition of low and high responders in the caption. Please avoid using red and green colors together in plots. The color coding also seems to vary randomly across the main figures.

Fig.2: Labels are too small. Panel A – What is shown? Individual worm responses are thin lines and broad lines with error bars are means with SEM? Is the data normally distributed? What is being summarized ? The response to the same stimulus, across species? If so, why aggregate across species?

Fig.3: It would be helpful to give each figure element a panel letter for clarity. For the traces, I suggest using time instead of frame number for the x-axis as frame is arbitrary. Briefly explain in the caption what the interpretation of the z-score plots is for the data shown. It would be helpful to have a schematic showing the James et al. protocol for context for B and C. Again, it seems like the data here was aggregated across species. Why?

**********

what does this mean?). If published, this will include your full peer review and any attached files.

If you choose “no”, your identity will remain anonymous but your review may still be made public.

**Do you want your identity to be public for this peer review?**  For information about this choice, including consent withdrawal, please see our Privacy Policy

Reviewer #1: **Yes:**  Danielle Ireland

Reviewer #2: No

**********

[NOTE: If reviewer comments were submitted as an attachment file, they will be attached to this email and accessible via the submission site. Please log into your account, locate the manuscript record, and check for the action link "View Attachments". If this link does not appear, there are no attachment files.]

To ensure your figures meet our technical requirements, please review our figure guidelines: https://journals.plos.org/plosone/s/figures

You may also use PLOS’s free figure tool, NAAS, to help you prepare publication quality figures: https://journals.plos.org/plosone/s/figures#loc-tools-for-figure-preparation.

NAAS will assess whether your figures meet our technical requirements by comparing each figure against our figure specifications.

# Author comment: Author response to Decision Letter 1

**DOI:** 10.1371/journal.pone.0358756.r003

13 Jul 2026

Please see "Response_to_Reviewers.docx" for specific responses to reviewer comments.

Attachment Submitted filename: Response_to_Reviewers.docx

# Peer review: Decision Letter 1

**DOI:** 10.1371/journal.pone.0358756.r004

**Authors:** Maria Y Pakharukova

10 Aug 2026

Dear Dr. Kelso,

Thank you for submitting your manuscript to PLOS ONE. After careful consideration, we feel that it has merit but does not fully meet PLOS ONE’s publication criteria as it currently stands. Therefore, we invite you to submit a revised version of the manuscript that addresses the points raised during the review process.

Please submit your revised manuscript by Sep 24 2026 11:59PM. If you will need more time than this to complete your revisions, please reply to this message or contact the journal office at plosone@plos.org. When you're ready to submit your revision, log on to https://www.editorialmanager.com/pone/ and select the 'Submissions Needing Revision' folder to locate your manuscript file.

- A letter that responds to each point raised by the academic editor and reviewer(s). You should upload this letter as a separate file labeled 'Response to Reviewers'.
- A marked-up copy of your manuscript that highlights changes made to the original version. You should upload this as a separate file labeled 'Revised Manuscript with Track Changes'.
- An unmarked version of your revised paper without tracked changes. You should upload this as a separate file labeled 'Manuscript'.

If you would like to make changes to your financial disclosure, please include your updated statement in your cover letter. Guidelines for resubmitting your figure files are available below the reviewer comments at the end of this letter.

If applicable, we recommend that you deposit your laboratory protocols in protocols.io to enhance the reproducibility of your results. Protocols.io assigns your protocol its own identifier (DOI) so that it can be cited independently in the future. For instructions see: https://journals.plos.org/plosone/s/submission-guidelines#loc-laboratory-protocols. Additionally, PLOS ONE offers an option for publishing peer-reviewed Lab Protocol articles, which describe protocols hosted on protocols.io. Read more information on sharing protocols at https://plos.org/protocols?utm_medium=editorial-email&utm_source=authorletters&utm_campaign=protocols.

As the corresponding author, your ORCID iD is verified in the submission system and will appear in the published article. PLOS supports the use of ORCID, and we encourage all coauthors to register for an ORCID iD and use it as well. Please encourage your coauthors to verify their ORCID iD within the submission system before final acceptance, as unverified ORCID iDs will not appear in the published article. *Only*  the individual author can complete the verification step; PLOS staff *cannot*  verify ORCID iDs on behalf of authors.

We look forward to receiving your revised manuscript.

Kind regards,

Maria Y Pakharukova, Ph.D., D.Sc.

Academic Editor

PLOS One

Journal Requirements:

1. If the reviewer comments include a recommendation to cite specific previously published works, please review and evaluate these publications to determine whether they are relevant and should be cited. There is no requirement to cite these works unless the editor has indicated otherwise.

2. Please review your reference list to ensure that it is complete and correct. If you have cited papers that have been retracted, please include the rationale for doing so in the manuscript text, or remove these references and replace them with relevant current references. Any changes to the reference list should be mentioned in the rebuttal letter that accompanies your revised manuscript. If you need to cite a retracted article, indicate the article’s retracted status in the References list and also include a citation and full reference for the retraction notice.

[Note: HTML markup is below. Please do not edit.]

Reviewers' comments:

Reviewer's Responses to Questions

**Comments to the Author**

Reviewer #1: (No Response)

Reviewer #2: (No Response)

**********

2. Is the manuscript technically sound, and do the data support the conclusions?

Reviewer #1: Yes

Reviewer #2: Yes

**********

3. Has the statistical analysis been performed appropriately and rigorously?

Reviewer #1: Yes

Reviewer #2: Yes

**********

4. Have the authors made all data underlying the findings in their manuscript fully available?

The PLOS Data policy

Reviewer #1: Yes

Reviewer #2: Yes

**********

5. Is the manuscript presented in an intelligible fashion and written in standard English?

Reviewer #1: Yes

Reviewer #2: Yes

**********

Reviewer #1: I appreciate the authors' efforts to incorporate the reviewers' feedback and think the paper is much improved. The authors have appropriately narrowed the scope of their conclusions to match the data presented; however, I believe it is still necessary to change the title accordingly, as previously mentioned by Reviewer 2, to make it clear which species were tested and that this failure is specific to specific testing protocols. Otherwise, I believe the manuscript is suitable for publication.

Reviewer #2: I appreciate the authors' careful and thorough response to the previous comments. The manuscript has been substantially strengthened and the revisions have improved its clarity and readability.

There are a few minor revisions that should be addressed prior to publication:

1) As mentioned previously, the title should explicitly name the planarian species that were tested

2) Please remove bioRxiv articles from the citations that have not yet undergone peer-review

3) Remove lines 291-292 from the Discussion "Because of our failure to demonstrate conditioned behavior, we

were not able to address questions of molecular transfer of memory between planarians in any way. " - a study of memory transfer was not within the scope of this work.

**********

what does this mean?). If published, this will include your full peer review and any attached files.

If you choose “no”, your identity will remain anonymous but your review may still be made public.

**Do you want your identity to be public for this peer review?**  For information about this choice, including consent withdrawal, please see our Privacy Policy

Reviewer #1: No

Reviewer #2: No

**********

[NOTE: If reviewer comments were submitted as an attachment file, they will be attached to this email and accessible via the submission site. Please log into your account, locate the manuscript record, and check for the action link "View Attachments". If this link does not appear, there are no attachment files.]

To ensure your figures meet our technical requirements, please review our figure guidelines: https://journals.plos.org/plosone/s/figures

You may also use PLOS’s free figure tool, NAAS, to help you prepare publication quality figures: https://journals.plos.org/plosone/s/figures#loc-tools-for-figure-preparation.

NAAS will assess whether your figures meet our technical requirements by comparing each figure against our figure specifications.

# Author comment: Author response to Decision Letter 2

**DOI:** 10.1371/journal.pone.0358756.r005

25 Aug 2026

Contents below are copied from Response_to_reviewers.docx.

----------------------------------------------------

PLOS One Manuscript ID: PONE-D-26-20336

Title: Failure to classically condition planarian flatworms (now updated to “Failure to classically condition G. dorotocephala and S. mediterranea flatworms using historical and contemporary light-shock paradigms”)

Response to Reviewers

We thank both reviewers for their continued feedback and have revised the manuscript in line with the remaining comments. Below we address all comments (the reviewer comments are shown in italics, followed by our responses). A PDF of the manuscript with tracked changes shown is attached.

Response to Reviewer 1

“I appreciate the authors' efforts to incorporate the reviewers' feedback and think the paper is much improved. The authors have appropriately narrowed the scope of their conclusions to match the data presented; however, I believe it is still necessary to change the title accordingly, as previously mentioned by Reviewer 2, to make it clear which species were tested and that this failure is specific to specific testing protocols. Otherwise, I believe the manuscript is suitable for publication.”

We thank the reviewer for this comment to further clarify the scope of the paper, and have updated the title to include the planarian species and conditioning paradigms used.

Response to Reviewer 2

“As mentioned previously, the title should explicitly name the planarian species that were tested.”

We thank the reviewer for their agreement to change the title, and have done as such (see above). We agree that this further clarifies the scope of the manuscript and claims.

“Please remove bioRxiv articles from the citations that have not yet undergone peer-review.”

We thank the reviewer for this comment and recognize the importance of restricting citations to published, peer-reviewed research. We have removed the BioRxiv citations of the Wyss et al. paper and have replaced the Samuel et al. citation with the peer-reviewed version published in 2026.

“Remove lines 291-292 from the Discussion "Because of our failure to demonstrate conditioned behavior, we were not able to address questions of molecular transfer of memory between planarians in any way. " - a study of memory transfer was not within the scope of this work.”

We understand the reviewer’s concern that this sentence distracts from the experiments presented in the paper, and upon rereading agree it isn’t suitable in the manuscript. The sentence has been removed.

Attachment Submitted filename: Response_to_reviewers_2nd_revision_Aug13.docx

# Editor report: Decision Letter 2

**DOI:** 10.1371/journal.pone.0358756.r006

**Authors:** Maria Y Pakharukova

7 Sep 2026

Failure to classically condition G. dorotocephala and S. mediterranea flatworms using historical and contemporary light-shock paradigms

PONE-D-26-20336R2

Dear Dr. Kelso,

We’re pleased to inform you that your manuscript has been judged scientifically suitable for publication and will be formally accepted for publication once it meets all outstanding technical requirements.

Within one week, you’ll receive an e-mail detailing the required amendments. When these have been addressed, you’ll receive a formal acceptance letter and your manuscript will be scheduled for publication.

An invoice will be generated when your article is formally accepted. Please note, if your institution has a publishing partnership with PLOS and your article meets the relevant criteria, all or part of your publication costs will be covered. Please make sure your user information is up-to-date by logging into Editorial Manager at Editorial Manager® and clicking the ‘Update My Information' link at the top of the page. For questions related to billing, please contact billing support.

If your institution or institutions have a press office, please notify them about your upcoming paper to help maximize its impact. If they’ll be preparing press materials, please inform our press team as soon as possible -- no later than 48 hours after receiving the formal acceptance. Your manuscript will remain under strict press embargo until 2 pm Eastern Time on the date of publication. For more information, please contact onepress@plos.org.

Kind regards,

Maria Y Pakharukova, Ph.D., D.Sc.

Academic Editor

PLOS One

Additional Editor Comments (optional):

Reviewers' comments:

# Editor report: Acceptance letter

**DOI:** 10.1371/journal.pone.0358756.r007

**Authors:** Maria Y Pakharukova

PONE-D-26-20336R2

PLOS One

Dear Dr. Kelso,

I'm pleased to inform you that your manuscript has been deemed suitable for publication in PLOS One. Congratulations! Your manuscript is now being handed over to our production team.

At this stage, our production department will prepare your paper for publication. This includes ensuring the following:

* All references, tables, and figures are properly cited

* All relevant supporting information is included in the manuscript submission,

* There are no issues that prevent the paper from being properly typeset

You will receive further instructions from the production team, including instructions on how to review your proof when it is ready. Please keep in mind that we are working through a large volume of accepted articles, so please give us a few days to review your paper and let you know the next and final steps.

Lastly, if your institution or institutions have a press office, please let them know about your upcoming paper now to help maximize its impact. If they'll be preparing press materials, please inform our press team within the next 48 hours. Your manuscript will remain under strict press embargo until 2 pm Eastern Time on the date of publication. For more information, please contact onepress@plos.org.

You will receive an invoice from PLOS for your publication fee after your manuscript has reached the completed accept phase. If you receive an email requesting payment before acceptance or for any other service, this may be a phishing scheme. Learn how to identify phishing emails and protect your accounts at https://explore.plos.org/phishing.

If we can help with anything else, please email us at customercare@plos.org.

Thank you for submitting your work to PLOS One and supporting open access.

Kind regards,

PLOS One Editorial Office Staff

on behalf of

Dr. Maria Y Pakharukova

Academic Editor

PLOS One
